# Implementing Structured Clinical Templates at a Single Tertiary Hospital: Survey Study

**DOI:** 10.2196/13836

**Published:** 2020-04-30

**Authors:** Ji Eun Hwang, Byung Ook Seoung, Sang-Oh Lee, Soo-Yong Shin

**Affiliations:** 1 Department of Digital Health Samsung Advanced Institute for Health Sciences & Technology Sungkyunkwan University Seoul Republic of Korea; 2 Office of Medical Information Asan Medical Center Seoul Republic of Korea; 3 Department of Infectious Diseases Asan Medical Center University of Ulsan College of Medicine Seoul Republic of Korea

**Keywords:** structured clinical template, structured data entry, data entry time, user experience, electronic health records

## Abstract

**Background:**

Electronic health record (EHR) systems have been widely adopted in hospitals. However, since current EHRs mainly focus on lowering the number of paper documents used, they have suffered from poor search function and reusability capabilities. To overcome these drawbacks, structured clinical templates have been proposed; however, they are not widely used owing to the inconvenience of data entry.

**Objective:**

This study aims to verify the usability of structured templates by comparing data entry times.

**Methods:**

A Korean tertiary hospital has implemented structured clinical templates with the modeling of clinical contents for the last 6 years. As a result, 1238 clinical content models (ie, body measurements, vital signs, and allergies) have been developed and 492 models for 13 clinical templates, including pathology reports, were applied to EHRs for clinical practice. Then, to verify the usability of the structured templates, data entry times from free-texts and four structured pathology report templates were compared using 4391 entries from structured data entry (SDE) log data and 4265 entries from free-text log data. In addition, a paper-based survey and a focus group interview were conducted with 23 participants from three different groups, including EHR developers, pathology transcriptionists, and clinical data extraction team members.

**Results:**

Based on the analysis of time required for data entry, in most cases, beginner users of the structured clinical templates required at most 70.18% more time for data entry. However, as users became accustomed to the templates, they were able to enter data more quickly than via free-text entry: at least 1 minute and 23 seconds (16.8%) up to 5 minutes and 42 seconds (27.6%). Interestingly, well-designed thyroid cancer pathology reports required 14.54% less data entry time from the beginning of the SDE implementation. In the interviews and survey, we confirmed that most of the interviewees agreed on the need for structured templates. However, they were skeptical about structuring all the items included in the templates.

**Conclusions:**

The increase in initial elapsed time led users to hold a negative opinion of SDE, despite its benefits. To overcome these obstacles, it is necessary to structure the clinical templates for optimum use. In addition, user experience in terms of ease of data entry must be considered as an essential aspect in the development of structured clinical templates.

## Introduction

### Background

The adoption rate of electronic health record (EHR) systems has increased dramatically [1,2]. However, since most physicians have been hesitant to change their behavior, most EHR systems have simply allowed conversion of paper documents into electronic documents by allowing free-text entries, similar to paper charts. These free-text entries led to multiple copying and pasting of the same content becoming common practice, blocking of the adoption of clinical decision support systems (CDSS), and making data extraction very difficult [3]. To overcome these drawbacks, two approaches have typically been applied: implementing structured clinical templates [4-6] for prospective data collection and applying natural language processing (NLP) [7-11] for retrospective data cleansing. The main focus of existing research is to apply clinical NLP techniques to clinical free-text templates [12-14]. However, though the importance and usability of these NLP approaches in various clinical documents have been demonstrated, they have mainly been used for the secondary usage of clinical data (ie, research purposes). To use CDSS in clinical practice, structured clinical templates should be implemented.

A substantial amount of effort and research has been applied by standardization communities to develop structured clinical templates (ie, EHR archetype [15-17], International Organization for Standardization [ISO] 13606 standard series [18-20], Clinical Information Modeling Initiative [CIMI] [21], and Clinical Element Models at Intermountain Healthcare [22-24]). Implementing standardized structured clinical templates can lead to diverse benefits, such as (1) preventing the use of different terms for the same meaning, (2) easily implementing CDSS, (3) easily extracting the necessary content from different templates, (4) preventing the re-entering of the same content, (5) helping to provide correct statistics and access to real-time statistics, and (6) reducing clinical errors and improving clinical outcomes. In short, the entire EHR template process, including development, management, and data extraction, can be improved [25-28]. In spite of these benefits, structured clinical templates are not popular in current EHRs owing to the inconvenience of data entry [29]. Structured data entry (SDE) in structured clinical templates is generally considered to take longer compared to free-text entry [30,31]. However, as far as we know [30], there is no detailed comparative analysis for data entry time between SDE and free-text. Although Trachtenbarg compared the elapsed time between free-text (ie, dictation) and discrete data (ie, SDE), there is a lack of data description [31]. Furthermore, in his study he conducted a comparison between handwriting and inputting data using the SDE. Here, we investigated the data entry time of SDEs and conducted a focus group interview based on 5 years’ experience with structured clinical templates and their application in a clinical practice. We also elucidated the important success factors for the adoption of structured clinical templates in EHRs.

### Objectives

In this study, we analyzed elapsed time while using SDE compared to free-text entries and performed a paper-based survey, using a 5-point Likert scale, regarding SDE. The patterns of elapsed time and the user survey data can be referenced by other medical institutions that want to build a structured EHR.

## Methods

### Clinical Template Selection

All clinical templates and data were chosen based on the needs of physicians. We also tried to select types of reports that were as diverse as possible by including reports that were only manually entered as well as those that included results automatically generated by medical devices. This helped confirm the possibility of the extension of structured clinical templates. The specific reasons for choosing the template are explained in Multimedia Appendix 1.

**Figure 1:**
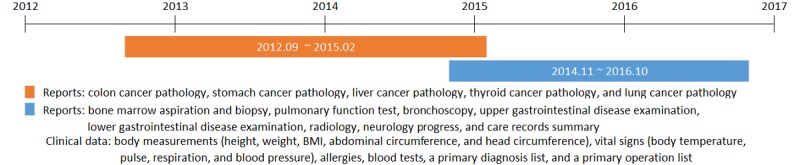
Timeline of structured data entry (SDE) development.

We developed structured clinical templates for use with the in-house EHR system of a tertiary hospital in Korea. Five types of pathology reports—colon cancer, stomach cancer, liver cancer, thyroid cancer, and lung cancer—were developed between September 2012 and February 2015 (see Figure 1). Next, eight reports were developed between November 2014 and October 2016; these reports were as follows: bone marrow aspiration and biopsy report, pulmonary function test report, bronchoscopy report, upper gastrointestinal disease examination report, lower gastrointestinal disease examination report, radiology report, neurology progress report, and care records summary (see Figure 1). During the same period, other clinical data were also standardized and structured; these data included the following: body measurements (ie, height, weight, BMI, abdominal circumference, and head circumference), vital signs (ie, body temperature, pulse, respiration, and blood pressure), allergies, blood tests, a primary diagnosis list, and a primary operation list (see Figure 1).

### Structured Clinical Template Development Methods

**Figure 2:**
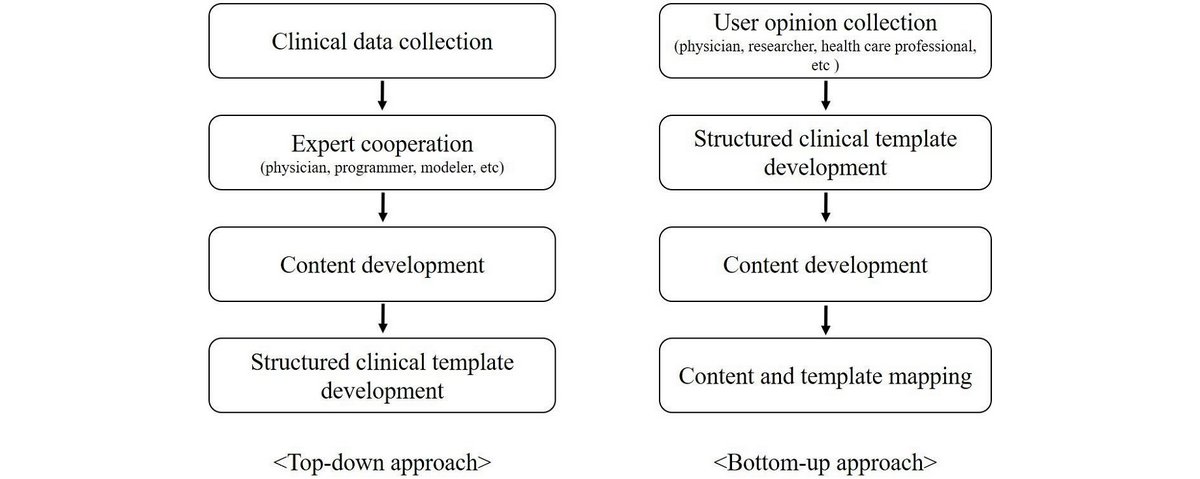
Comparison between top-down approach and bottom-up approach.

A decade ago, the Korean Research and Development Center for Interoperable EHR developed a structured clinical template development guide [32]. It is a top-down approach: centralized management collected all relevant data, and then content development was carried out through expert collaboration. After that, the structured clinical template was designed based on developed content. However, implementing the structured clinical template based on the above guide was too time-consuming and required laborious work, owing to its top-down approach. To implement the structured clinical templates within this study’s limited time frame, we combined top-down and bottom-up approaches when implementing the templates, as shown in Figure 2. The bottom-up approach, as opposed to the top-down method, approaches the design of the structured template by consulting physicians first. We discussed the design with users who routinely entered data for those reports to clarify necessary data models; we also discussed the design with researchers, including physicians, who use the input data, since we do not necessarily need to model all data in the clinical notes. The SDE for the template was then designed, considering the input of physicians. At this stage, there was no model for clinical data; the SDE was just a user interface. After designing the SDE interface, clinical contents were modeled and mapped to it. Therefore, the clinical models might not be comprehensive but, rather, curated for only the target template. Finally, a viewer form was also implemented, since SDE is not suitable for viewing purposes. The detailed comparison between the top-down and the bottom-up approaches is shown in Multimedia Appendix 2.

The templates, which consist of only numbers and codes, were implemented using a top-down approach since they can easily be modeled. The note formats, such as for the pathology reports and progress notes, were implemented using a bottom-up approach. With both of these approaches, the SDE was implemented using an in-house template designer. The data entered in an SDE template are stored in the XML format and in relational database format in Oracle Database. The developed content models were controlled by the institutional committee, and they have been reused and updated.

### Data Entry Time Log Collection

**Figure 3:**
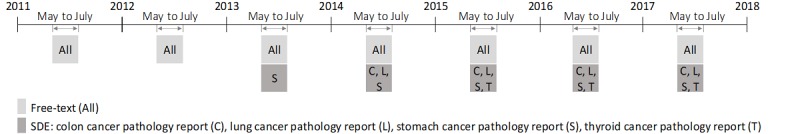
Summary of the data entry time log collection. SDE: structured data entry.

To compare the data entry time, we collected medical transcriptionists’ data entry times for four pathology reports—stomach, lung, colon, and thyroid cancer—because these reports contained enough log data. We collected the log data of SDEs from the deployment of each SDE through 2017, as well as the free-text log data from 2011 to 2017. Log data were collected from SDEs for stomach cancer pathology reports from 2013 to 2017, lung cancer and colon cancer reports from 2014 to 2017, and thyroid cancer reports from 2015 to 2017. Specifically, log data timestamped from May to July (ie, 3 months) were collected for each year. The timestamps collected were between 8 am and 6 pm each day, excluding the lunch break (noon-1 pm). If the total elapsed time of a single report exceeded 1 hour, the data were discarded as outlying. Figure 3 shows a summary of information about the data entry time log collection.

Transcriptionists’ work was carried out by choosing a sample number, inputting the contents, and storing the data. Thus, we employed an operational definition of elapsed time by subtracting the saving time from the selecting time.

### Statistical Analysis

The data of elapsed time did not follow a normal distribution, so we conducted nonparametric analyses. To determine whether there were differences in the types of data entry times, we used the Wilcoxon rank-sum test. We also used the Kruskal-Wallis test and the Wilcoxon rank-sum test to compare the elapsed time between the first year of SDE, SDE in 2017, and free-text. For statistical analysis, we used the software program R, version 3.6.1 (The R Foundation).

After applying SDEs, we surveyed three groups on different topics. All the questionnaires were different between the groups, so the comparison of scores between the groups was not meaningful. However, we conducted parametric analyses because the data extraction team survey results between 2013 and 2017 did follow a normal distribution. We used two-sample *t* tests using R, version 3.6.1 (The R Foundation).

## Results

### Overview

We developed 1238 content models and 13 templates using 1129 entities, 385 qualifiers, 1583 value sets, and 5664 values. Some entities, value sets, and values were reused from the previous models. More detailed information on the number of the developed entities, qualifiers, value sets, and values are explained in Multimedia Appendix 3. We also included the figures that are part of the thyroid SDE template and the thyroid cancer data entry interface in Multimedia Appendix 4 and Multimedia Appendix 5, respectively. As the appendix figures show, the SDE consists of drop-down lists, single check boxes, duplicate check boxes, and so forth.

### Data Entry Time for Pathology Structured Data Entry

Table 1 shows a comparison of the median data entry time for free-text and SDEs for each type of pathology report. For free-text, the data entry times were the median value from 2011 through the year of the initial SDE deployment. For SDE, the data entry times were the median value from the year of the initial deployment through 2017. The detailed log data for each year are shown in Multimedia Appendix 6. Stomach cancer SDE required the longest data entry time compared to free-text (ie, 2 minutes and 34 seconds). However, colon cancer SDE and thyroid cancer SDE required less time than free-text entry (ie, 2 minutes and 26 seconds, and 2 minutes and 12 seconds, respectively).

Table 2 shows a detailed comparison of the results between the first year of SDE deployment and 2017 (ie, the year in which users grew accustomed to the use of SDEs after several years of experience) and free-text entries. The total entry time for SDEs is taken as the middle-most value of a single year (ie, the first year or 2017) and that for free-text is the same as in Table 1. For stomach cancer pathology reports, which required the most data entry time, the SDE took longer, with an increase of 6 minutes and 33 seconds (70.18%). However, thyroid cancer SDEs saw a reduction in the data entry time from the first year by 1 minute and 38 seconds (14.54%). Even the elapsed time for the thyroid cancer report SDE, which required the least data entry time compared to free-text, decreased (3 minutes and 1 second, 31.42%). For stomach cancer reports, the data entry time decreased dramatically, by 5 minutes and 5 seconds (47.07%), from 2013 to 2017. Though reduced time to enter data for colon cancer was not proved to be statistically significant, in all cases users were able to enter data using SDE faster than with free-text after several years of experience.

As in Multimedia Appendix 6, each SDE shows different variations of data entry times. Data entry time for thyroid cancer SDE has steadily decreased since SDE was implemented. Other SDEs, such as stomach cancer, lung cancer, and colon cancer, showed alternating increases and decreases in the elapsed time. In particular, it is interesting that in the second year of SDE implementation, for lung cancer, data entry time increased by 10.92% (87 seconds) compared to the first year. A new method of lung cancer surgery was introduced in 2015, the second year of SDE implementation. This led to an increase in the number of collected specimens and pathologic examination items. In addition, factors such as the number of entries that must be entered owing to regulation changes have also affected the data entry time. However, overall, data entry time has decreased as users have become more familiar with SDEs.

**Table 1:** Comparison of elapsed time between structured data entry (SDE) and free-text for pathology reports.

Report	Free-text			SDE			Entry time comparison (free-text – SDE), min:sec^a^	*P* value
	Entry time, min:sec	Total number of reports, n	Year, range	Entry time, min:sec	Total number of reports, n	Year, range		
Stomach cancer	9:20	1096	2011-2012	11:54	1373	2013-2017	+2:34	<.001
Lung cancer	12:07	661	2011-2013	12:46	729	2014-2017	+0:39	.05
Colon cancer	13:10	945	2011-2013	10:44	1289	2014-2017	–2:26	<.001
Thyroid cancer	11:14	1563	2011-2014	9:02	970	2015-2017	–2:12	<.001

^a^Minutes and seconds.

**Table 2:** Comparison of elapsed time between the first year of structured data entry (SDE), SDE in 2017, and free-text.

Report	Free-text		SDE: first year of deployment		SDE: 2017	Entry time comparison, min:sec^a^ (% rate of change)		
	Entry time (A), min:sec	Year, range	Entry time (B), min:sec	Year	Entry time (C), min:sec	A vs B	A vs C	B vs C
Stomach cancer	9:20	2011-2012	15:53	2013	10:48	+6:33 (+70.18)^b^	+1:28 (+9.23)^b^	–5:05 (–47.07)^b^
Lung cancer	12:07	2011-2013	13:17	2014	11:16	+1:10 (+9.63)^b^	–0:51 (–6.40)	–2:01 (–17.90)^b^
Colon cancer	13:10	2011-2013	11:21	2014	10:55	–1:49 (–13.80)	–2:15 (–19.82)^b^	–0:26 (–4.00)
Thyroid cancer	11:14	2011-2014	9:36	2015	8:13	–1:38 (–14.54)^b^	–3:01 (–31.42)^b^	–1:23 (–16.80)^b^

^a^Minutes and seconds.

^b^*P*<.05.

### Interview Results

**Figure 4:**
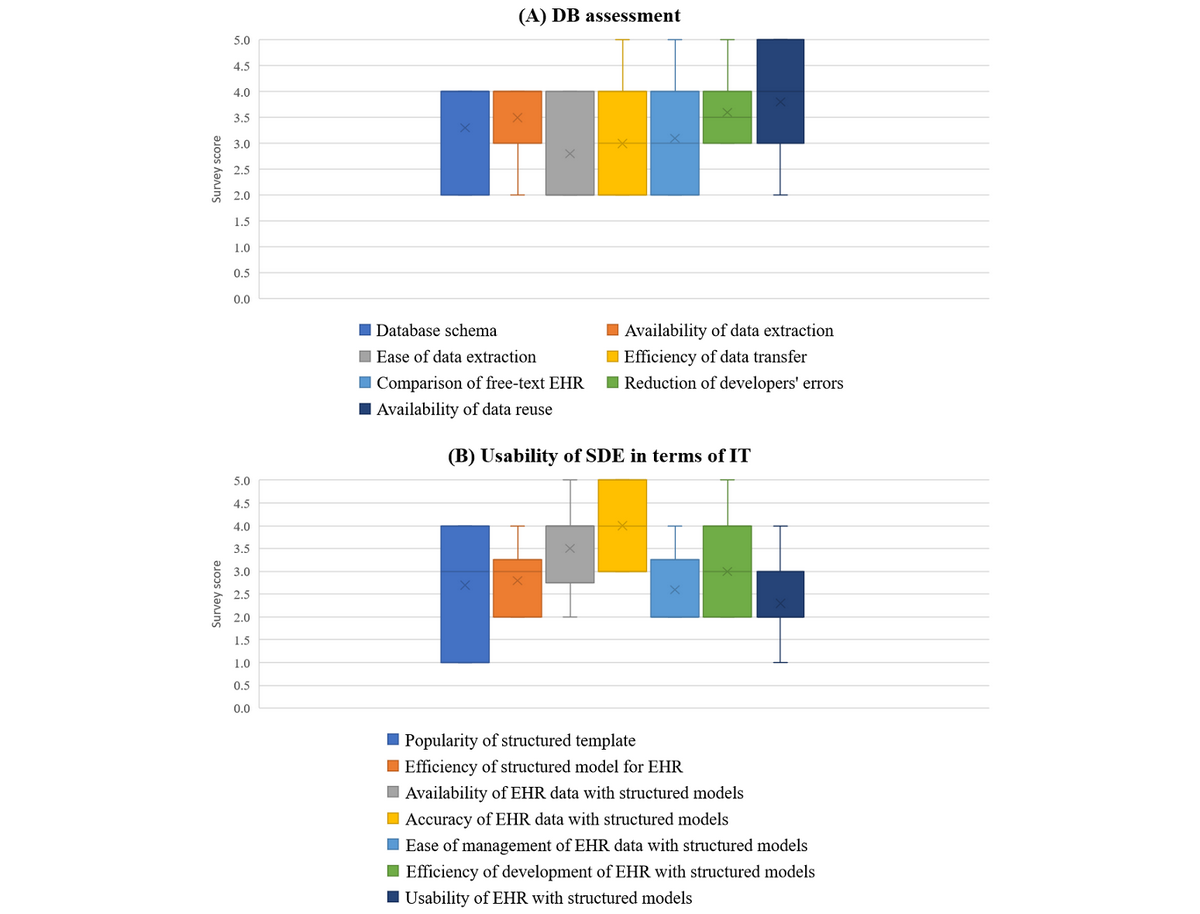
Survey results from the electronic health record (EHR) developer team. Survey scores range from 1 (strongly disagree) to 5 (strongly agree). DB: database; IT: information technology; SDE: structured data entry.

To verify the merits of EHRs with structured models, we performed a paper-based survey and a focus group interview with three different groups: an EHR developer team, a pathology transcriptionist team, and a research data extraction team. The paper-based survey included questions on a 5-point Likert scale, ranging from 1 (strongly disagree) to 5 (strongly agree). The EHR developer team consisted of 10 in-house EHR developers. As shown in Figure 4 (A), the developers gave high scores (3.3 points), on average, for database assessment. *Availability of data reuse* received the highest score, as expected. *Ease of data extraction* received the lowest score owing to more complicated database queries. However, since this structured EHR requires the consideration of the parent-child relationship of clinical content models when developing templates, the *usability of EHRs with structured models* received the lowest score, as shown in Figure 4 (B). On the other hand, *accuracy of EHR data with structured models* received the highest score (4.0 points).

The EHR developer focus group interview results indicated that developers agreed with the merits of EHRs with structured templates due to data reusability. Interestingly, they felt that EHRs with structured templates can improve and standardize the process of EHR template development and reduce the overheads of EHR system management. Before adopting the structured templates, if the term in a specific template is changed, all the templates which contain the same term should be changed manually. However, by using content models in the structured template, this process can be automated. The developers worried about the overhead of EHR development caused by the complicated structure and process of structured templates. Therefore, to reduce this development overhead, only the necessary models should be developed, and the simple Entity-Value (EV) structure should be widely used, rather than the complicated Entity-Qualifier-Value (EQV) structure.

**Figure 5:**
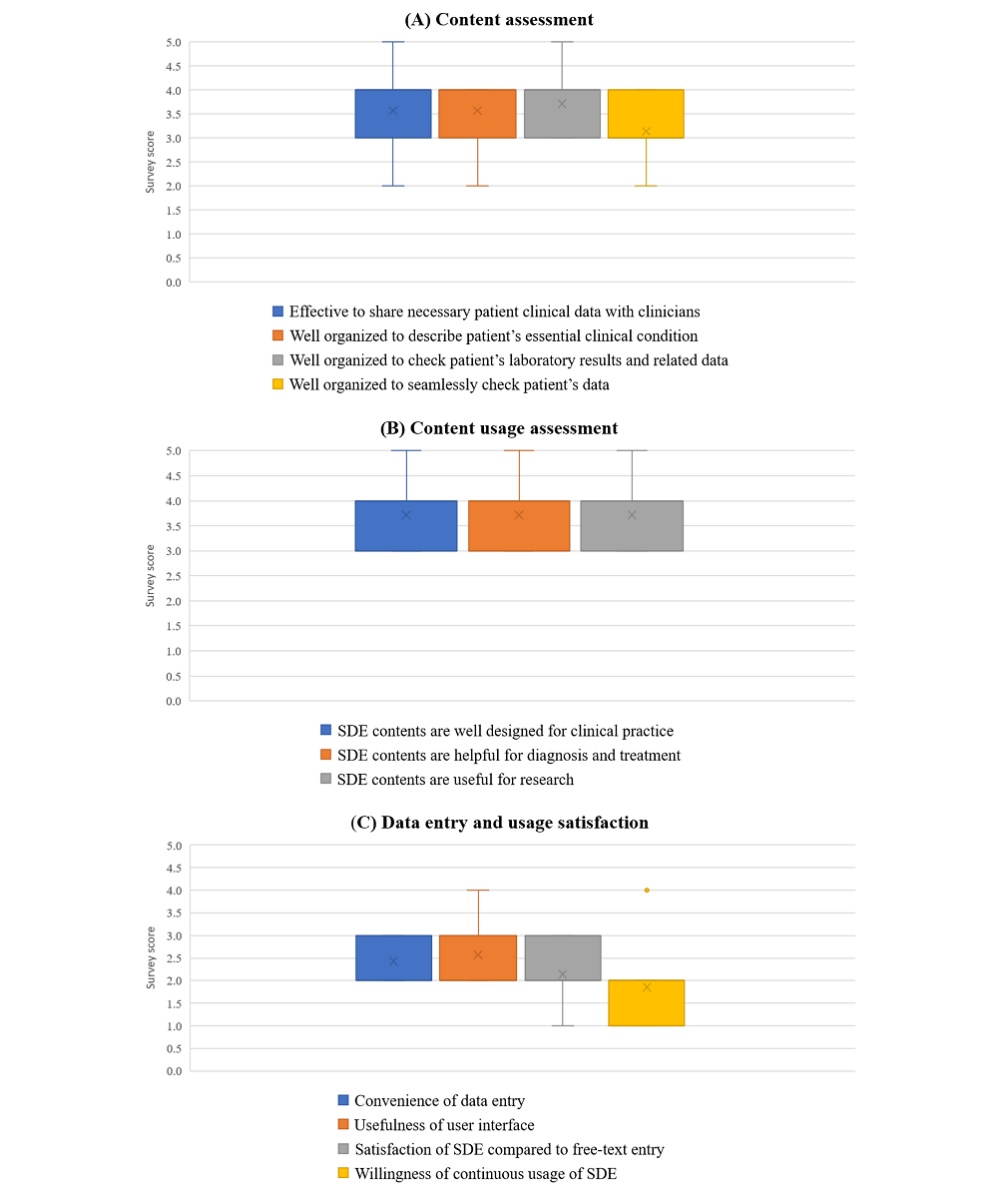
Survey results from the pathology transcriptionists. Survey scores range from 1 (strongly disagree) to 5 (strongly agree). SDE: structured data entry.

The second focus group consisted of seven pathology transcriptionists who filled in the content of the templates based on an interpretation of pathologists’ verbal notes. They valued the content of the structured clinical templates, as shown in Figure 5. However, because of the longer data entry time, they ultimately did not want to use structured clinical templates (1.86 points). One user, however, approved of the use of structured templates despite the longer data entry time because this approach benefited all users.

**Figure 6:**
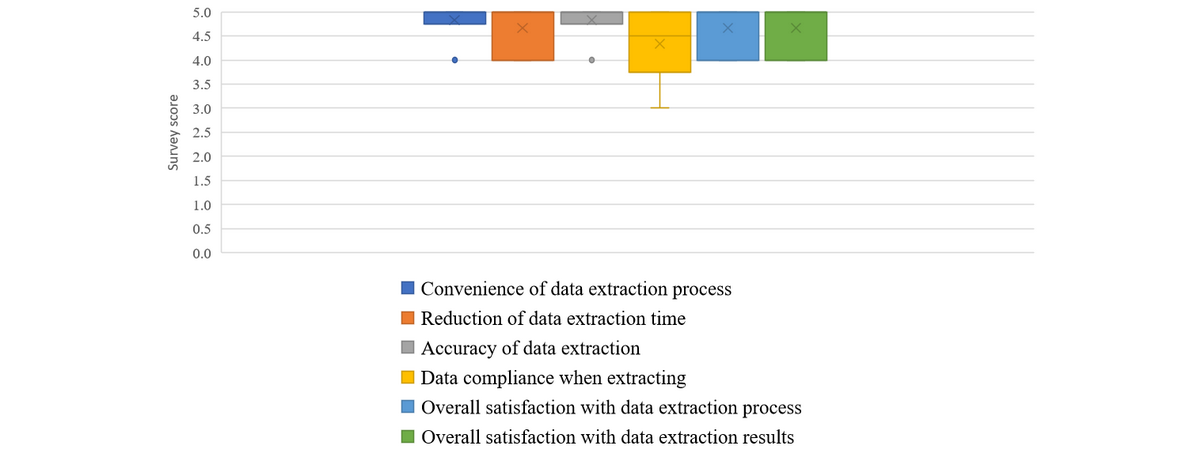
Survey results, regarding data extraction of structured data entries (SDEs), from the data extraction team in 2017. Survey scores range from 1 (strongly disagree) to 5 (strongly agree).

The third focus group consisted of six research data extraction team members. The team consisted of two programmers, two registered nurses, and two health information managers. On average, they had more than 4 years’ experience with data extraction from EHRs. They preferred the structured clinical templates in all aspects, such as *convenience of data extraction process*, *reduction of data extraction time*, *accuracy of extracted data*, *missing data*, and *overall satisfaction with structured data entry* (see Figure 6).

**Figure 7:**
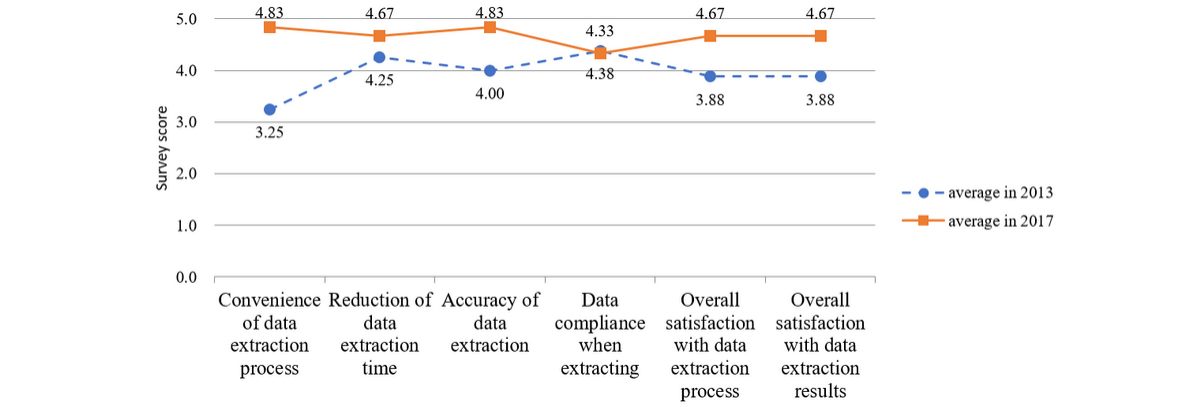
Comparison of survey results, regarding data extraction of structured data entries (SDEs), from the data extraction team between 2013 and 2017. Scores range from 1 (strongly disagree) to 5 (strongly agree).

Since this team performed the same interview in 2013 when initially implementing structured clinical templates, we compared the survey results (see Figure 7). The differences in scores for *reduction of data extraction time* and *data compliance when extracting* were not statistically significant. However, the average scores increased significantly, from 3.94 to 4.67. Interestingly, *data compliance when extracting*, which was rated highest in 2013, was rated lowest in 2017. In the interviews, the participants noted that the exact data entry depends on the users, not on the structured data entry process. Though a few structured clinical templates were used in EHRs in 2013, the overall satisfaction rate increased significantly. It should be mentioned that the 2013 survey results may have been based on the expectations of structured clinical template usage, while the 2017 survey results were based on actual practical experience. This implies that the data extraction team was satisfied with the structured clinical template beyond their original expectations. However, structured data entry does not solve data incompleteness problems, since SDE was mainly developed to increase the ease of data entry, not necessarily data usage.

## Discussion

### Principal Findings

To utilize the clinical data in EHRs, structured clinical templates are essential. However, the adoption rate of SDE was low. Among the diverse obstructive factors for the adoption of SDE, we focused on data entry time, since many users complained that it took much longer compared to free-text templates. On reviewing previous studies, we found that Trachtenbarg mentioned that “clicking or typing text multiple times is generally slower than dictating” [31]. We must mention that Trachtenbarg’s study compared the data entry of SDEs and handwritten text, not free-text using a keyboard. Therefore, we can conclude that the hypothesis of this study, namely, “using structured templates requires more data entry time compared to free-text” is supported.

Many physicians stated that when they conducted research, they experienced the problems of low-quality data, a lot of missing values, and inconsistent data, among other issues. Physicians expect that SDE will help facilitate their research [33]. Therefore, to encourage users to use SDE, we emphasized that SDE can facilitate research. In many cases, the same entities were included in different SDEs. In the unstructured data entry format, repeated typing results in inconsistency and incompleteness and is time-consuming, while in SDE, the data entered in a previous SDE are automatically filled in to other SDEs [34-36]. As users will not be allowed to save the template if they do not enter all the required fields, SDEs force the users to enter all the required entities and ensure completeness [37,38].

We also provided convenience in the terminology used, by adopting automatic word completion as in Google Web searches. In addition, we adopted the interface terminology server, and users can freely enter the necessary terms registered in it. The terms of the interface terminology server are mapped to the reference terminology, such as SNOMED CT (Systematized Nomenclature of Medicine Clinical Terms) and LOINC (Logical Observation Identifiers Names and Codes), and we tried to allow users the freedom to choose familiar words. The terminology server has representative terms and the diverse variations of each, which have the same meaning, are internally mapped to a representative term. Therefore, users can use diverse terms if desired.

Typically, the development of structured clinical templates begins with designing basic clinical models and then implementing SDEs. For thorough coverage of a clinical model, this top-down approach is required. However, this approach requires very long implementation times. For this study, we designed the SDE first, and then the necessary clinical models for the SDE items were developed. In addition, we did not implement all items as EQV models. Many items were implemented as EV models. As in the Agile model in the information technology area [39], implementing and then revising the model is necessary to reflect user requirements and to reduce development time. Clinicians can formulate an idea when using the templates; thus, a simple approach is beneficial. However, all clinical models for SDEs were precoordinated for ease of data entry. This bottom-up approach can save a substantial amount in terms of development costs, but it has the disadvantage of model granularity. The models are developed based on the SDE, and while some models can have detailed meanings, others can have very abstract ones. This bottom-up approach is still, however, a practical method since (1) models can be developed with a small number of physicians and modelers and (2) this method can guarantee an easy user interface.

To reduce the data entry time for SDEs, there are two important considerations: (1) minimizing structured components and (2) using input patterns suitable for SDE. For example, the colon cancer SDE has only the minimum necessary components based on previous experience, and the thyroid cancer template already had a standardized input pattern, which is helpful when implementing SDE.

The data extraction team was satisfied with the implemented structured clinical templates. It is possible that this satisfaction was mainly based on the hospital’s clinical data warehouse, especially because the clinical data warehouse can easily be improved to support structured templates, and so the team can easily extract the data. This group also noticed that the quality of the data was not related to structured templates. If SDE restricts more and more data entries as mandatory input, users will resist the use of SDE owing to its inconvenience. Therefore, when developing SDE, the balance between data usefulness and user convenience should be considered. For example, SDE for thyroid cancer requires less data entry time than free-text templates. A well-designed SDE and choice of proper templates are essential. In addition, although users initially required more data entry time with SDEs, the required time decreased as they became accustomed to SDE use.

Our hypothesis was proven through applying SDE to cancers, especially stomach cancer pathology reports and lung cancer pathology reports. We also developed diverse structured templates, such as admission note, discharge note, and nursing record, as described in the Methods section. In our experience, there is no significant difference between cancer, noncancer, and other reports. We reported the analysis results of the cancer pathology reports, since these reports contain many reusable data and are easy to structure. In addition, there are commissioned items on these reports. We hope that cancer pathology reports can be easily adopted in other hospitals.

The limitation of this study is that we did not adopt a solid usability method. TURF (Toward a Unified Framework of EHR Usability) is a well-known usability framework [40]. If we had applied solid usability studies such as TURF, our hypothesis would have been more powerful. However, the templates we developed were part of a next-generation EHR system to upgrade the entire hospital information system. In addition, the questionnaires were used to determine the satisfaction of users with the new hospital information system. This means that this study was not designed for research purposes using a rigorous scientific framework but, rather, for business practices. In addition, for various reasons, due to item changes, such as a change in government policy, advancements in medical science, different annual numbers of patients, and unbalanced data, we could not conduct stringent statistical analyses. However, we did calculate median values and *P* values using nonparametric tests. Thus, we think that our study will help other hospitals, because most other medical institutions are in a similar situation where they do not have enough time, manpower, and finances. Our study’s findings emphasize that usability is a key element to the successful implementation of SDE.

### Conclusions

Currently, EHRs are typically simply word processors, as they focus only on the digitization of clinical data. For the next generation of EHRs, a spreadsheet-style approach rather than a word processor-style approach should be implemented. This requires the structuralization of the data.

As far as we know, this is the first study to analyze elapsed data entry time in a real clinical setting. Previously, only user surveys had been conducted to explore elapsed time for SDE. Through this study, we were able to confirm that SDEs usually require more time than free-text entries. This time-consuming effort hinders SDE adoption despite the many benefits of structured clinical templates. Therefore, when designing SDE, the focus should be on the reduction of data entry time to achieve successful deployment. As in the case of colon and thyroid cancer, well-optimized and well-designed SDE will reduce the elapsed data entry time. Therefore, it is also necessary to select an item to be structured from all the template items. We also confirmed that the data entry time for SDE decreases as users become accustomed to using the templates, leading to SDE ultimately requiring less time than free-text entry. To overcome the initial time-consuming efforts, research on user experience should be carried out to reduce the data entry time burden of SDE.

## Appendix

Multimedia Appendix 1 Structured target templates and items.

Multimedia Appendix 2 Top-down approach and bottom-up approach to implementing structured data entry (SDE).

Multimedia Appendix 3 Summary of developed models.

Multimedia Appendix 4 Thyroid pathology structured data entry (SDE) template.

Multimedia Appendix 5 Thyroid cancer data entry interface.

Multimedia Appendix 6 The detailed log data from 2011 through 2017 for four pathology reports.

## Abbreviations

CDSS: clinical decision support system
CIMI: Clinical Information Modeling Initiative
EHR: electronic health record
EQV: Entity-Qualifier-Value
EV: Entity-Value
ISO: International Organization for Standardization
LOINC: Logical Observation Identifiers Names and Codes
MOTIE: Ministry of Trade, Industry, and Energy
NLP: natural language processing
SDE: structured data entry
SNOMED CT: Systematized Nomenclature of Medicine Clinical Terms
TURF: Toward a Unified Framework of EHR Usability

## References

[1] (2018). Office of the National Coordinator for Health Information Technology.

[2] Kim Y, Jung K, Park Y, Shin D, Cho SY, Yoon D, Park RW (2017). Rate of electronic health record adoption in South Korea: A nation-wide survey. Int J Med Inform.

[3] Berger ML, Curtis MD, Smith G, Harnett J, Abernethy AP (2016). Opportunities and challenges in leveraging electronic health record data in oncology. Future Oncol.

[4] Mamlouk MD, Chang PC, Saket RR (2018). Contextual radiology reporting: A new approach to neuroradiology structured templates. AJNR Am J Neuroradiol.

[5] Shaish H, Feltus W, Steinman J, Hecht E, Wenske S, Ahmed F (2018). Impact of a structured reporting template on adherence to Prostate Imaging Reporting and Data System version 2 and on the diagnostic performance of prostate MRI for clinically significant prostate cancer. J Am Coll Radiol.

[6] Bink A, Benner J, Reinhardt J, De Vere-Tyndall A, Stieltjes B, Hainc N, Stippich C (2018). Structured reporting in neuroradiology: Intracranial tumors. Front Neurol.

[7] Sung S, Chen K, Wu DP, Hung L, Su Y, Hu Y (2018). Applying natural language processing techniques to develop a task-specific EMR interface for timely stroke thrombolysis: A feasibility study. Int J Med Inform.

[8] Goff DJ, Loehfelm TW (2018). Automated radiology report summarization using an open-source natural language processing pipeline. J Digit Imaging.

[9] Huhdanpaa HT, Tan WK, Rundell SD, Suri P, Chokshi FH, Comstock BA, Heagerty PJ, James KT, Avins AL, Nedeljkovic SS, Nerenz DR, Kallmes DF, Luetmer PH, Sherman KJ, Organ NL, Griffith B, Langlotz CP, Carrell D, Hassanpour S, Jarvik JG (2018). Using natural language processing of free-text radiology reports to identify Type 1 Modic endplate changes. J Digit Imaging.

[10] Fonferko-Shadrach B, Lacey A, Akbari A, Thompson S, Ford D, Lyons R, Rees M, Pickrell O (2018). Using natural language processing to extract structured epilepsy data from unstructured clinic letters. Int J Popul Data Sci.

[11] Sabra S, Alobaidi M, Malik K, Sabeeh V (2018). Performance evaluation for semantic-based risk factors extraction from clinical narratives. Proceedings of the IEEE 8th Annual Computing and Communication Workshop and Conference.

[12] Gonzalez-Hernandez G, Sarker A, O'Connor K, Savova G (2017). Capturing the patient's perspective: A review of advances in natural language processing of health-related text. Yearb Med Inform.

[13] Luo Y, Thompson WK, Herr TM, Zeng Z, Berendsen MA, Jonnalagadda SR, Carson MB, Starren J (2017). Natural language processing for EHR-based pharmacovigilance: A structured review. Drug Saf.

[14] Yim W, Yetisgen M, Harris WP, Kwan SW (2016). Natural language processing in oncology: A review. JAMA Oncol.

[15] Oliveira D, Coimbra A, Miranda F, Abreu N, Leuschner P, Machado J (2018). New approach to an openEHR introduction in a Portuguese healthcare facility. Proceedings of the 6th World Conference on Information Systems and Technologies (WorldCIST'18).

[16] Mascia C, Uva P, Leo S, Zanetti G (2018). OpenEHR modeling for genomics in clinical practice. Int J Med Inform.

[17] Pedersen R, Granja C, Marco-Ruiz L (2017). Implementation of openEHR in combination with clinical terminologies: Experiences from Norway. Int J Adv Life Sci.

[18] Kopanitsa G, Taranik M (2015). Application of ISO 13606 archetypes for an integration of hospital and laboratory information systems. Proceedings of the 21st International Conference on Information and Software Technologies (ICIST 2015).

[19] Santos MR, Bax MP, Kalra D (2010). Building a logical EHR architecture based on ISO 13606 standard and semantic Web technologies. Stud Health Technol Inform.

[20] Moner D, Maldonado JA, Angulo C, Bosca D, Perez D, Abad I, Reig E, Robles M (2008). Standardization of discharge reports with the ISO 13606 norm. Conf Proc IEEE Eng Med Biol Soc.

[21] Sharma DK, Solbrig HR, Prud'hommeaux E, Pathak J, Jiang G (2016). Standardized representation of clinical study data dictionaries with CIMI archetypes. AMIA Annu Symp Proc.

[22] Lee J, Hulse NC, Wood GM, Oniki TA, Huff SM (2016). Profiling Fast Healthcare Interoperability Resources (FHIR) of family health history based on the clinical element models. AMIA Annu Symp Proc.

[23] Zhu Q, Freimuth RR, Pathak J, Chute CG (2013). Using clinical element models for pharmacogenomic study data standardization. AMIA Jt Summits Transl Sci Proc.

[24] Oniki TA, Zhuo N, Beebe CE, Liu H, Coyle JF, Parker CG, Solbrig HR, Marchant K, Kaggal VC, Chute CG, Huff SM (2016). Clinical element models in the SHARPn consortium. J Am Med Inform Assoc.

[25] Schoeppe F, Sommer WH, Haack M, Havel M, Rheinwald M, Wechtenbruch J, Fischer MR, Meinel FG, Sabel BO, Sommer NN (2018). Structured reports of videofluoroscopic swallowing studies have the potential to improve overall report quality compared to free text reports. Eur Radiol.

[26] Lin E, Powell DK, Kagetsu NJ (2014). Efficacy of a checklist-style structured radiology reporting template in reducing resident misses on cervical spine computed tomography examinations. J Digit Imaging.

[27] Marcovici PA, Taylor GA (2014). Journal Club: Structured radiology reports are more complete and more effective than unstructured reports. AJR Am J Roentgenol.

[28] Weiss DL, Langlotz CP (2008). Structured reporting: Patient care enhancement or productivity nightmare?. Radiology.

[29] Sahni VA, Silveira PC, Sainani NI, Khorasani R (2015). Impact of a structured report template on the quality of MRI reports for rectal cancer staging. AJR Am J Roentgenol.

[30] Ganeshan D, Duong PT, Probyn L, Lenchik L, McArthur TA, Retrouvey M, Ghobadi EH, Desouches SL, Pastel D, Francis IR (2018). Structured reporting in radiology. Acad Radiol.

[31] Trachtenbarg DE (2007). EHRs fix everything--and nine other myths. Fam Pract Manag.

[32] Ahn S (2010). Development and application of development principles for clinical information model. J Korea Acad Ind Coop Soc.

[33] Häyrinen K, Saranto K, Nykänen P (2008). Definition, structure, content, use and impacts of electronic health records: A review of the research literature. Int J Med Inform.

[34] Hu S, Yen DH, Kao W (2002). The feasibility of full computerization in the ED. Am J Emerg Med.

[35] Porcheret M, Hughes R, Evans D, Jordan K, Whitehurst T, Ogden H, Croft P, North Staffordshire General Practice Research Network (2004). Data quality of general practice electronic health records: The impact of a program of assessments, feedback, and training. J Am Med Inform Assoc.

[36] Tange HJ, Hasman A, de Vries Robbé PF, Schouten HC (1997). Medical narratives in electronic medical records. Int J Med Inform.

[37] Cheung NT, Fung V, Chow YY, Tung Y (2001). Structured data entry of clinical information for documentation and data collection. Stud Health Technol Inform.

[38] Ho LM, McGhee SM, Hedley AJ, Leong JC (1999). The application of a computerized problem-oriented medical record system and its impact on patient care. Int J Med Inform.

[39] Martin RC (2002). Agile Software Development: Principles, Patterns, and Practices.

[40] Zhang J, Walji MF (2011). TURF: Toward a unified framework of EHR usability. J Biomed Inform.

