# Supplementary material for: Implementing Structured Clinical Templates at a Single Tertiary Hospital: Survey Study
Source: JMIR Med Inform. 2020 Apr 30;8(4):e13836. doi: 10.2196/13836 (PMC7226057; doi:10.2196/13836)
Supplement: Multimedia Appendix 1 [file medinform_v8i4e13836_app1.pdf]

Multimedia Appendix 1. Structured target templates and items.

When we developed clinical template models, user needs were the first priority. Then, in view of the lack of time, we chose the target template for them. The specific reasons for choosing the template are explained in the table below.

		Reason for structured templates	Period of development
Pathology reports	Colon cancer	Manually entered report	2014–2015
	Stomach cancer		
	Liver cancer		
	Thyroid cancer		
	Lung cancer		
Bone marrow aspiration and biopsy report		Clinician request	2015
Pulmonary function test report		Manually entered report	2015
Bronchoscopy report		Automatically entered report from medical device	2015
Primary diagnosis list		Key information for clinical statistics	2015
Body measurements	Height	Essential information of clinical observation	2016
	Weight		
	BMI		
	Abdominal circumference		
	Head circumference		
Vital signs	Body temperature	Essential information of clinical observation	2016
	Pulse		
	Respiration		
	Blood pressure		
Allergies	Adverse drug reactions	Essential information of clinical observation	2016
Primary operation list		Key information on the operation record	2016
Blood tests		The most useful among laboratory results	2016
Gastrointestinal disease examination reports	Upper gastrointestinal disease examination report	High percentage of total number tests	2016
	Lower gastrointestinal disease examination report		
Radiology report	Brain magnetic resonance imaging	Extension of structured template	2016

	(MRI) report	to MRI report	
Neurology progress report	Stroke record	Reduce missing entries	2016
Care records summary		Use for health information exchanges	2016
