# Supplementary material for: Implementing Structured Clinical Templates at a Single Tertiary Hospital: Survey Study
Source: JMIR Med Inform. 2020 Apr 30;8(4):e13836. doi: 10.2196/13836 (PMC7226057; doi:10.2196/13836)
Supplement: Multimedia Appendix 2 [file medinform_v8i4e13836_app2.pdf]

Multimedia Appendix 2. Top-down approach and bottom-up approach to implementing structured data entry (SDE).

We used two approaches to develop the structured clinical template: 1) top-down, and 2) bottom-up. The table below shows the detailed comparison between the top-down and bottom-up approach.

	Top-down approach	Bottom-up approach
Concept	General to specific<br>Centralized control	Specific to general<br>Distributed management
Method	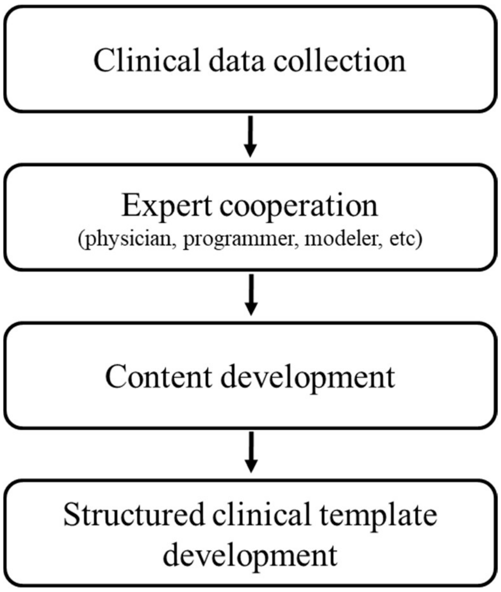	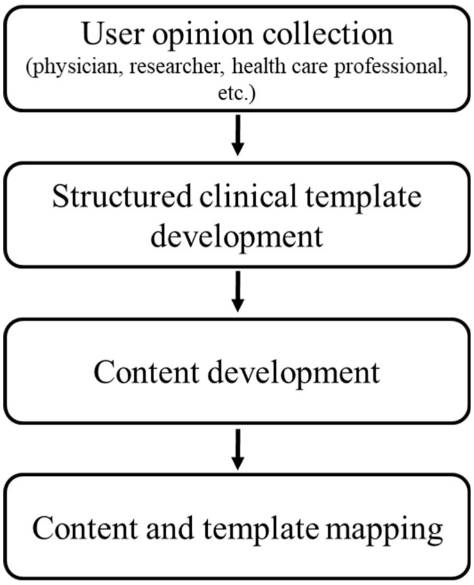
Strength	Accurate modeling	Short development period
Weakness	Time-consuming<br>Required laborious work	Granularity
Implement	Body measurements, vital signs, blood tests, etc.	Note formats (pathology reports, progress notes)
