# Supplementary material for: Implementing Structured Clinical Templates at a Single Tertiary Hospital: Survey Study
Source: JMIR Med Inform. 2020 Apr 30;8(4):e13836. doi: 10.2196/13836 (PMC7226057; doi:10.2196/13836)
Supplement: Multimedia Appendix 3 [file medinform_v8i4e13836_app3.pdf]

Multimedia Appendix 3. Summary of developed models.

We developed 1,238 content models and 13 templates using 1,129 entities, 385 qualifiers, 1,583 value sets, and 5,664 values. Some entities, value sets, and values were reused from the previous models. More detailed information on the numbers of developed entity, qualifier, value set, and values is presented in the table below.

		Number of elements	Number of mappings[a]
Pathological report of colon cancer	Entity	82	18
	Qualifier	36	21
	Value Set	58	39
	Value	165	99
Pathological report of stomach cancer	Entity	77	18
	Qualifier	32	22
	Value Set	43	31
	Value	137	67
Pathological report of liver cancer	Entity	57	28
	Qualifier	46	22
	Value Set	67	43
	Value	184	96
Pathological report of thyroid cancer	Entity	33	15
	Qualifier	22	13
	Value Set	26	17
	Value	85	45
Pathological report of lung cancer	Entity	85	21
	Qualifier	32	21
	Value Set	38	26
	Value	138	78
Bone marrow aspiration and biopsy report	Model	110	
	Entity	105	75
	Qualifier	42	22
	Value Set	46	31
	Value	228	135
Pulmonary function test report	Model	63	
	Entity	63	41
	Qualifier	8	7
	Value Set	2	0
	Value	5	5
Bronchoscopy report	Model	22	
	Entity	19	10
	Qualifier	38	27
	Value Set	21	15
	Value	90	53
Primary diagnosis list	Model	520	
	Entity	520	508
	Qualifier	110	102

	Value Set	1136	200
	Value	3402	3166
Body measurements	Model	6	
	Entity	6	2
	Qualifier	10	10
	Value Set	11	10
	Value	63	58
Vital signs	Model	9	
	Entity	6	6
	Qualifier	15	14
	Value Set	20	13
	Value	100	99
Allergies	Model	11	
	Entity	11	0
	Qualifier	8	10
	Value Set	20	14
	Value	238	232
Primary operation list	Model	50	
	Entity	50	50
	Qualifier	37	30
	Value Set	72	44
	Value	482	429
Blood tests	Model	106	
	Entity	106	106
	Qualifier	28	22
	Value Set	99	5
	Value	39	37
Gastrointestinal disease examination report	Model	50	
	Entity	40	26
	Qualifier	60	51
	Value Set	67	33
	Value	315	229
Radiology report (Brain MRI)	Model	21	
	Entity	16	14
	Qualifier	21	13
	Value Set	16	12
	Value	67	65
Neurology progress report	Model	50	
	Entity	44	35
	Qualifier	60	48
	Value Set	66	29
	Value	260	219
	Value	315	229
Care records summary	Model	19	
	Entity	19	11
	Qualifier	84	61

	Value Set	32	16
	Value	341	153

[a] SNOMED CT and LOINC were used.
