# Supplementary material for: Implementing Structured Clinical Templates at a Single Tertiary Hospital: Survey Study
Source: JMIR Med Inform. 2020 Apr 30;8(4):e13836. doi: 10.2196/13836 (PMC7226057; doi:10.2196/13836)
Supplement: Multimedia Appendix 4 [file medinform_v8i4e13836_app4.pdf]

Multimedia Appendix 4. Thyroid pathology structured data entry (SDE) template.

The figure below is a part of the thyroid pathology SDE template. As the figure shows, the SDE template consists of drop-down list, single check boxes, duplicate check boxes, etc.

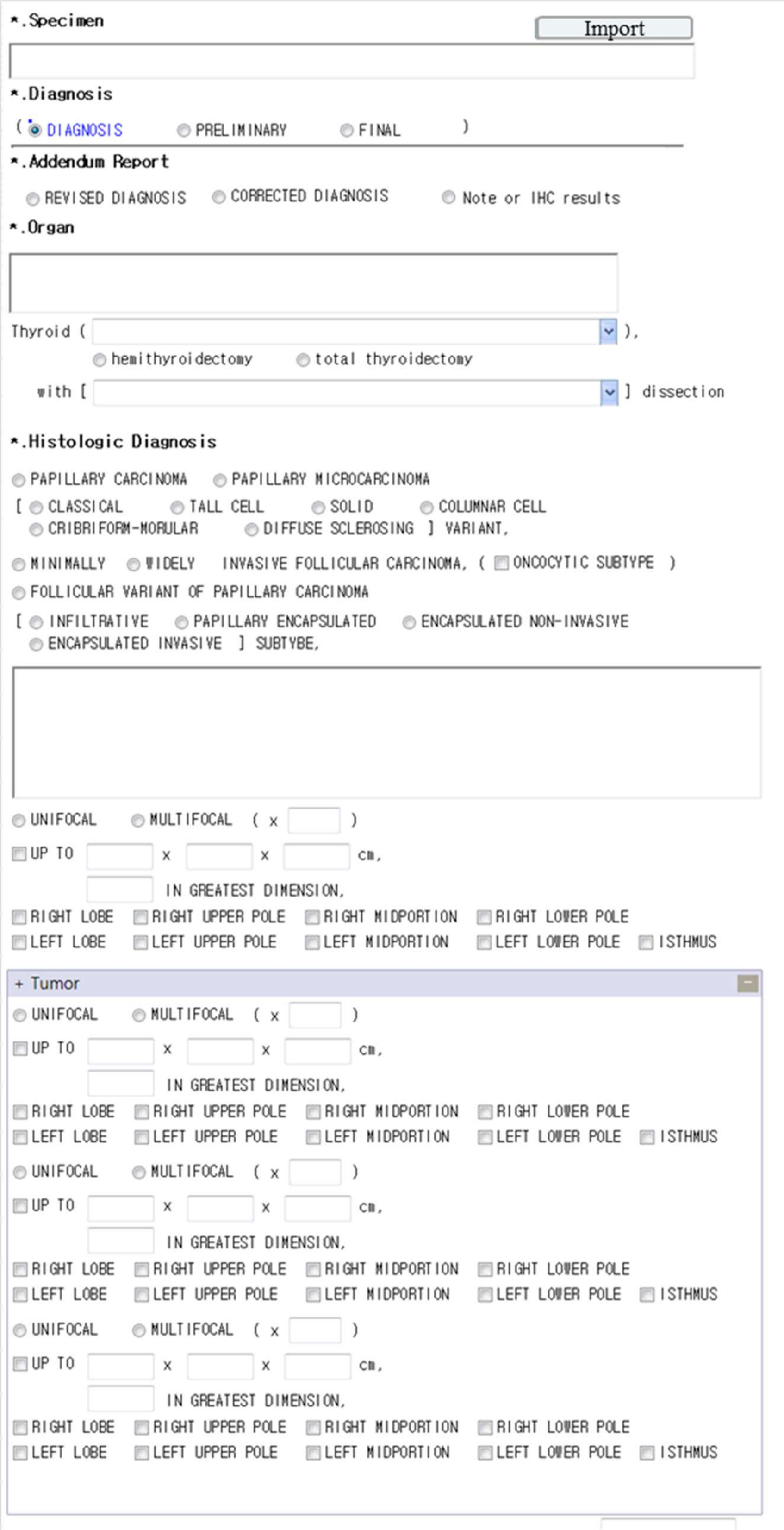

*.Specimen

Import

*.Diagnosis

(DIAGNOSIS PRELIMINARY FINAL)

*.Addendum Report

REVISED DIAGNOSIS CORRECTED DIAGNOSIS Note or IHC results

*.Organ

Thyroid ( ),

hemithyroidectomy total thyroidectomy

with [ ] dissection

*.Histologic Diagnosis

PAPILLARY CARCINOMA PAPILLARY MICROCARCINOMA

[CLASSICAL TALL CELL SOLID COLUMNAR CELL

CRIBRIFORM-MORULAR DIFFUSE SCLEROSING] VARIANT,

MINIMALLY WIDELY INVASIVE FOLLICULAR CARCINOMA, (ONCOCYTIC SUBTYPE)

FOLLICULAR VARIANT OF PAPILLARY CARCINOMA

[INFILTRATIVE PAPILLARY ENCAPSULATED ENCAPSULATED NON-INVASIVE

ENCAPSULATED INVASIVE] SUBTYBE,

UNIFOCAL MULTIFOCAL (x)

UP TO x x cm,

IN GREATEST DIMENSION,

RIGHT LOBE RIGHT UPPER POLE RIGHT MIDPORTION RIGHT LOWER POLE

LEFT LOBE LEFT UPPER POLE LEFT MIDPORTION LEFT LOWER POLE ISTHMUS

+ Tumor

UNIFOCAL MULTIFOCAL (x)

UP TO x x cm,

IN GREATEST DIMENSION,

RIGHT LOBE RIGHT UPPER POLE RIGHT MIDPORTION RIGHT LOWER POLE

LEFT LOBE LEFT UPPER POLE LEFT MIDPORTION LEFT LOWER POLE ISTHMUS

UNIFOCAL MULTIFOCAL (x)

UP TO x x cm,

IN GREATEST DIMENSION,

RIGHT LOBE RIGHT UPPER POLE RIGHT MIDPORTION RIGHT LOWER POLE

LEFT LOBE LEFT UPPER POLE LEFT MIDPORTION LEFT LOWER POLE ISTHMUS

UNIFOCAL MULTIFOCAL (x)

UP TO x x cm,

IN GREATEST DIMENSION,

RIGHT LOBE RIGHT UPPER POLE RIGHT MIDPORTION RIGHT LOWER POLE

LEFT LOBE LEFT UPPER POLE LEFT MIDPORTION LEFT LOWER POLE ISTHMUS
