# Supplementary material for: Implementing Structured Clinical Templates at a Single Tertiary Hospital: Survey Study
Source: JMIR Med Inform. 2020 Apr 30;8(4):e13836. doi: 10.2196/13836 (PMC7226057; doi:10.2196/13836)

Multimedia Appendix 5. Thyroid cancer data entry interface.

The figure below is a part of the thyroid cancer data entry interface.

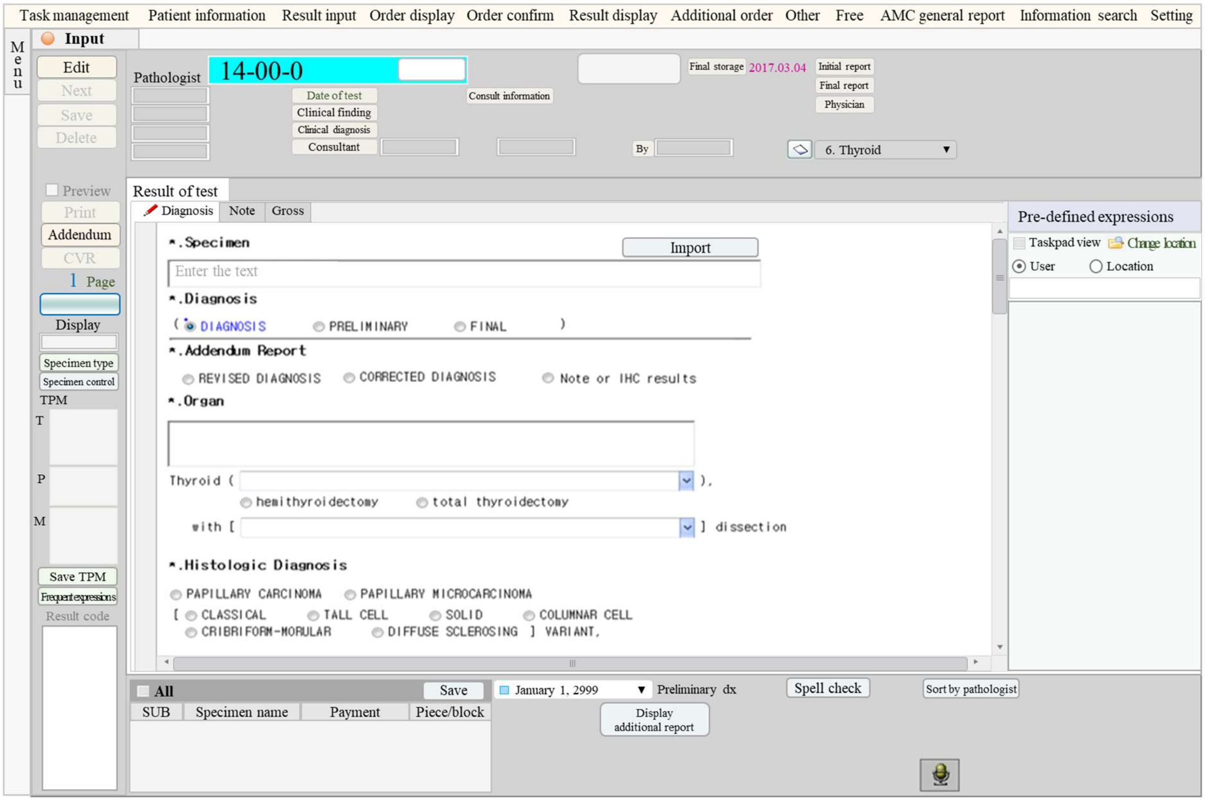

Supplement: Multimedia Appendix 5 [file medinform_v8i4e13836_app5.pdf]
