# Supplementary material for: Implementing Structured Clinical Templates at a Single Tertiary Hospital: Survey Study
Source: JMIR Med Inform. 2020 Apr 30;8(4):e13836. doi: 10.2196/13836 (PMC7226057; doi:10.2196/13836)
Supplement: Multimedia Appendix 6 [file medinform_v8i4e13836_app6.pdf]

Multimedia Appendix 6. The detailed log data from 2011 through 2017 for four pathology reports.

A comparison of the median data entry time for free-text and SDEs for each type of pathology report was carried out. The four tables below show the detailed log data for each year and each group of pathology reports.

(A) Stomach cancer report

Year	SDE		Free-text	
	Number of reports	Median elapsed time	Number of reports	Median elapsed time
2011	N/A	N/A	575	09 min 20 sec
2012	N/A	N/A	521	09 min 21 sec
2013	271	15 min 53 sec	34	07 min 05 sec
2014	281	12 min 02 sec	24	07 min 16 sec
2015	293	11 min 48 sec	19	11 min 13 sec
2016	282	09 min 52 sec	35	07 min 12 sec
2017	276	10 min 48 sec	42	06 min 46 sec

(B) Lung cancer report

Year	SDE		Free-text	
	Number of reports	Median elapsed time	Number of reports	Median elapsed time
2011	N/A	N/A	200	11 min 17 sec
2012	N/A	N/A	231	10 min 19 sec
2013	N/A	N/A	230	14 min 07 sec
2014	151	13 min 17 sec	59	06 min 51 sec
2015	163	14 min 44 sec	66	09 min 56 sec
2016	204	11 min 45 sec	56	08 min 55 sec
2017	211	11 min 16 sec	62	08 min 41 sec

(C) Colon cancer report

Year	SDE		Free-text	
	Number of reports	Median elapsed time	Number of reports	Median elapsed time
2011	N/A	N/A	361	12 min 55 sec
2012*	157	14 min 20 sec	238	12 min 05 sec
2013	N/A	N/A	346	14 min 36 sec
2014	304	11 min 21 sec	57	09 min 19 sec
2015	320	10 min 46 sec	48	09 min 46 sec
2016	354	10 min 11 sec	65	10 min 55 sec
2017	311	10 min 55 sec	59	10 min 08 sec

* Template change. Except for SDE cases in 2012 for fair comparison

(D) Thyroid cancer report

Year	SDE		Free-text	
	Number of reports	Median elapsed time	Number of reports	Median elapsed time
2011	N/A	N/A	396	10 min 51 sec
2012	N/A	N/A	413	11 min 20 sec
2013	N/A	N/A	403	12 min 20 sec
2014	N/A	N/A	351	10 min 39 sec
2015	303	09 min 36 sec	11	09 min 45 sec
2016	380	09 min 12 sec	5	05 min 10 sec
2017	287	08min 13 sec	15	09 min 42 sec
